# A native chromatin immunoprecipitation (ChIP) protocol for studying histone modifications in strawberry fruits

**DOI:** 10.1186/s13007-020-0556-z

**Published:** 2020-02-03

**Authors:** Xiaorong Huang, Qinwei Pan, Ying Lin, Tingting Gu, Yi Li

**Affiliations:** 1 State Key Laboratory of Plant Genetics and Germplasm Enhancement and College of Horticulture, Nanjing Agricultural University, Nanjing, 210095 People’s Republic of China; 2 Minzhulu Primary School of Xuzhou City, Xuzhou, 221000 People’s Republic of China; 3 Department of Plant Science and Landscape, Architecture, University of Connecticut, Storrs, CT 06269 USA

**Keywords:** Chromatin immunoprecipitation, Native ChIP, Fruit tissues, Strawberry, Histone modification

## Abstract

**Background:**

Covalent modifications of histones and histone variants have great influence on chromatin structure, which is involved in the transcriptional regulation of gene expression. Chromatin immunoprecipitation (ChIP) is a powerful tool for studying in vivo DNA-histone interactions. Strawberry is a model for Rosaceae and non-climacteric fruits, in which histone modifications have been implicated to affect fruit development and ripening. However, a validated ChIP method has not been reported in strawberry, probably due to its high levels of polysaccharides which affect the quality of prepared chromatin and the efficiency of immunoprecipitation.

**Results:**

We describe a native chromatin immunoprecipitation (N-ChIP) protocol suitable for strawberry by optimizing the parameters for nuclei isolation, chromatin extraction, DNA fragmentation and validation analysis using quantitative real-time PCR (qRT-PCR). The qRT-PCR results show that both the active mark H3K36me3 and the silent mark H3K9me2 are efficiently immunoprecipitated for the enriched regions. Compared to X-ChIP (cross-linked chromatin followed by immunoprecipitation), our optimized N-ChIP procedure has a higher signal-to-noise ratio and a lower background for both the active and the silent histone modifications. Furthermore, high-throughput sequencing following N-ChIP demonstrates that nearly 90% of the enriched H3K9/K14ac peaks are overlapped between biological replicates, indicating its remarkable consistency and reproducibility.

**Conclusions:**

An N-ChIP method suitable for the fleshy fruit tissues of woodland strawberry *Fragaria vesca* is described in this study. The efficiency and reproducibility of our optimized N-ChIP protocol are validated by both qRT-PCR and high-throughput sequencing. We conclude that N-ChIP is a more suitable method for strawberry fruit tissues relative to X-ChIP, which could be combined with high-throughput sequencing to investigate the impact of histone modifications in strawberry and potentially in other fruits with high content of polysaccharides.

## Background

Chromatin is composed of DNAs, histones, chromosomal proteins and associated RNAs [1–3]. Nucleosome is the fundamental unit of chromatin, which consists of a histone octamer and ~ 147 bp DNA wrapped around. Histone modifications on the N-terminal tails of core histones play an important role in epigenetic regulation, which greatly influences chromatin structures and gene expressions [4, 5].

Chromatin immunoprecipitation (ChIP) is widely used for profiling DNA–protein interactions in vivo*.* ChIP is a powerful method that allows one to identify the specific genomic regions associated with a protein of interest. With the appropriate antibodies, it can be used to locate histones carrying specific covalent modifications, such as acetylation, phosphorylation, or methylation.

X-ChIP (cross-linked chromatin followed by immunoprecipitation) and N-ChIP (native chromatin immunoprecipitation) are the two most commonly used ChIP methods. In X-ChIP, chromatin is cross-linked by formaldehyde, then sheered by sonication or enzymes for fragmentation [6]. While in N-ChIP, chromatin is isolated without cross-linking, and micrococcal nuclease (MNase) is used to digest the linker DNA between nucleosomes in native chromatin state [7–9]. In both cases, fragmented chromatin is immunoprecipitated by a specific antibody recognizing the protein of interest and then DNA is isolated for further analyses. ChIP followed by microarrays or high-throughput sequencing builds a genome-wide profiling of histone modifications, which provides information for the construction of chromatin structure and the possible regulatory roles of epigenetic factors [10–12]. For most non-histone chromosomal proteins, X-ChIP is the only option as they are not retained on the DNA during nuclease digestion of native chromatin. However, N-ChIP performs better for profiling histones and histone modifications in terms of the better antibody specificity, higher pull-down efficiency, lower background and less bias toward open chromatin [13, 14].

The diploid woodland strawberry *Fragaria vesca* with an assembled genome is emerging as a model plant for Rosaceae species and non-climacteric fruits [15]. Recently, it is reported that epigenetic factors such as histone modifications may be essential for fruit development and ripening in strawberry [16]. ChIP protocols have been developed in *Arabidopsis* and other plant species for non-fleshy-fruit tissues [17, 18]. However, ChIP protocols available in other species may not work efficiently in non-tested species. Compared to Arabidopsis leaf, root, flower or dry fruit tissues, strawberry fleshy fruits are characterized as higher water content and higher levels of polysaccharides and other secondary metabolites, which may lead to low yield of chromatin and reduced efficiency of the immunoprecipitation (IP). A validated ChIP method with high reproducibility for strawberry fruit has not been reported.

The principles of an N-ChIP protocol include the extraction of clean nuclei, proper fragmentation of native chromatin into single nucleosomes, efficient immunoprecipitation with the antibodies of interest, purification of immune complexes after immunoprecipitation, and analysis of bound DNA by quantitative PCR or sequencing [13, 14]. In this study, we describe an N-ChIP method with several adjustments based on some existing protocols [19–21] for some of the steps listed above. Thus, the protocol we used is not brand new in its principles but takes into account the features inherent to strawberry fleshy fruits such as their high content of water and polysaccharides. The antibodies against the silent histone mark H3K9me2 or the active histone marks H3K36me3 and H3K9/K14ac were used for immunoprecipitation. Quantitative real-time PCR (qRT-PCR) data validate the quality of chromatin preparation and the efficiency of immunoprecipitation, and suggest our N-ChIP as a better protocol for strawberry fruits compared to X-ChIP. Furthermore, this N-ChIP protocol produces highly consistent ChIP-seq data among biological replicates, indicating that it is suitable for profiling the genome-wide distribution of histone modifications in fleshy fruits.

## Methods

### Plant materials

**Fig. 1:**
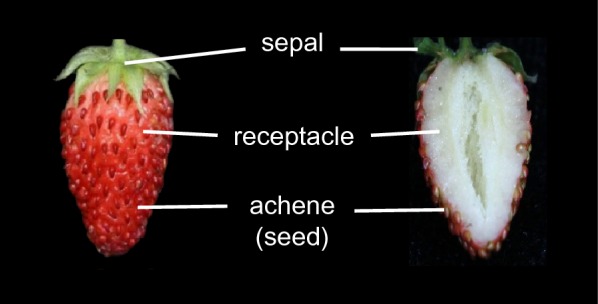
The red-stage strawberry fruits used in this study. The receptacles (fleshy fruits without seeds) were used for chromatin preparations

Plants of diploid woodland strawberry *Fragaria vesca* (Rugen4–7) were grown in a greenhouse at 16 h/8 h (light/dark), 65% relative humidity and 22 °C. Strawberry fruits at the red stage (about 1 month after anthesis, or 2 days after the pink stage) were collected (Fig. 1). Fresh fruits used for N-ChIP were immediately frozen in liquid nitrogen after harvesting. Fresh fruits used for X-ChIP were first fixed by 1% formaldehyde for 10 min and then immediately put into liquid nitrogen for further nuclei isolation.

### Reagents for N-ChIP

Sucrose (Sigma, cat. No. S9378).

Sodium chloride (Sigma, cat. No. S7653).

Deoxycholic acid, sodium salt (Sigma, cat. No. D6750).

Potassium chloride (Sigma, cat. No. P9541).

β-mercaptoethanol (Solarbio, cat. No. M8210).

Hexylene glycol (Sigma, cat. No.68340-500ML).

Magnesium chloride (Sigma, cat. No. M8266).

Proteinase K (20 mg ml^−1^) (Fermentas, cat. No. EO0491).

Triton X-100 (Sigma, cat. No. T8787).

Tris Base (Sigma, cat. No. 08656).

Glycine (Sigma, cat. No. G7403).

Ethanol (GHTECH, cat. No.1.17113.023).

Calcium chloride (Sigma, cat. No. C2661).

DL-Dithiothreitol (DTT) (Solarbio, cat. No. D8220).

PMSF (Sigma, cat. No. P7626-5G).

cOmplete™, Mini Protease Inhibitor Cocktail (Roche, cat. No. 04693159001).

EDTA disodium salt (Sigma, cat. No. E5134).

SDS (Sigma, cat. No. L6026).

Potassium Hydrogen phosphate Anhydrous (GHTECH, cat. No. 1.01938.010).

Potassium Phosphate Monobasic (GHTECH, cat. No. 1.01863.020).

Sodium Acetate (Sigma, cat. No. S2889).

Glycogen (Thermo Fisher, R0561).

Phenol/chloroform/isoamyl alcoholl (25:24:1) (Solarbio, cat. No.P1012).

Dynabeads® Protein A (Thermo Fisher, cat. No 10001D).

SYBR Premix Ex Tag (Takara).

### Additional reagents for X-ChIP

Formaldehyde 37% (sigma, cat. No. F8775).

### Antibodies for immumoprecipitation

Anti-dimethyl-histone H3 (Lys 9) (Abcam, ab1220).

Anti-trimethyl-histone H3 (Lys 27) (Abcam, ab6002).

Anti-acrtyl-histone H3 (Lys 9/Lys14) (Millipore, 07-329).

### Equipment for N-ChIP

Miracloth (Millipore, cat. No. 478588-1R).

Vortexer (SCIENTIFIC INDUSTRIES, G560E).

Freezers (Xuehua, IMS-50).

Water bath (Hualida, HS-800D).

Glass homogenizer (KIMBLE CHASE, 885303–0040).

PureProteome magnetic stand (MiniMACS2™ Separator, 130-042-102).

Applied Biosystems 7500 real-time PCR system (Novland, Shanghai, China).

### Additional equipments for X-ChP

Vacuum chamber (Vacuubrand, ME2CNT).

Bioruptor (UCD-600TS).

### Reagent setup

Always freshly prepare the buffers using stock solutions on the day of use and keep them on ice until required. PMSF, DTT, β-mercaptoethanol and protease inhibitor cocktail (PI) should be added into the solutions just before use.

Extraction buffer 1: 10 mM Potassium Phosphate (pH 7.0), 100 mM NaCl, 11.8% (v/v) Hexylene glycol, 11 mM β-mercaptoethanol, 1 mM PMSF, 1 mM DTT.

Extraction buffer 2: 10 mM Potassium Phosphate (pH 7.0), 100 mM NaCl, 10 mM MgCl_2_, 11.8% (v/v) Hexylene glycol, 11 mM β-mercaptoethanol, 1 mM PMSF, 1 mM DTT.

Micrococcal nuclease buffer (MNB): 0.3 M Sucrose, 50 mM Tris–HCl (pH 7.5), 4 mM MgCl_2_, 1 mM CaCl_2_.

Lysis buffer: 1 mM Tris–HCl (pH7.5), 1 mM PMSF, 1 mM DTT, 1 × PI.

Incubation buffer: 50 mM NaCl, 20 mM Tris–HCl (pH7.5), 5 mM EDTA, 1 mM PMSF, 1 × PI.

Low salt wash buffer: 50 mM Tris–HCl (pH 7.5), 10 mM EDTA, 50 mM NaCl.

Medium salt wash buffer: 50 mM Tris–HCl (pH 7.5), 10 mM EDTA, 100 mM NaCl.

High salt wash buffer: 50 mM Tris–HCl (pH 7.5), 10 mM EDTA, 150 mM NaCl.

Elution buffer: 50 mM NaCl, 20 mM Tris–HCl (pH 7.5), 5 mM EDTA, 1% SDS.

### Procedure of N-ChIP for strawberry fruits

Perform all the following steps at 4 °C. Keep the samples and precool all the solutions on ice unless otherwise indicated.

### Nuclei isolation

1. Use two grams of the red-stage strawberry fruits without seeds as the starting material.
2. Grind the fruits in liquid nitrogen to a fine powder with a pre-chilled mortar and pestle. Keep the sample frozen in liquid nitrogen to prevent it from thawing during grinding.
3. Transfer the fine powder into a pre-chilled 50 ml falcon tube and suspend the sample by 40 ml of PBS. Vortex to mix.
4. Spin at 1500* g* for 10 min at 4 °C and decant the supernatant.
5. Repeat step 3–4.
6. Re-suspend the pellet in 40 ml of extraction buffer 1. Vortex to mix, then homogenize in a pre-chilled glass homogenizer by using pre-chilled pestles (10 times with pestle A, 10 times with pestle B).
7. Keep the homogenized tissues on ice for 10 min with gentle agitation to have complete homogenization.
8. Filter the homogenate through two layers of miracloth into a new pre-chilled 50 ml Falcon tube.
9. Centrifuge at 1500* g* for 10 min at 4 °C.
10. Discard the supernatant and re-suspend the pellet in 15 ml of extraction buffer 2.
11. Repeat step 9–10 at least four times to collect clean nuclei.
12. Re-suspend the pellet in 3 ml of micrococcal nuclease buffer followed by centrifugation at 1500*g* for 10 min at 4 °C.
13. Discard the supernatant completely and add 250 μl of micrococcal nuclease buffer to gently re-suspend the pellet for micrococcal nuclease digestion (collect about 400 μl nuclei at this step).

### Chromatin fragmentation

1. Incubate the nuclei with 1 μl of micrococcal nuclease (MNase) for 10 min in a water bath set at 37 °C. Gently invert the tubes every 2 or 3 min during incubation (before this, appropriate digestion time needs to be determined for each chromatin preparation.)
2. Transfer the tubes into ice box and add 20 μl of 0.5 M EDTA to each tube to stop the reaction.
3. Centrifuge the tubes at 13,000* g* for 10 min at 4 °C.
4. Transfer the supernatant (S1, about 250 μl each tube) into a new 5 ml Eppendorf tube on ice. Re-suspend the pellet in 250 μl of lysis buffer. Leave the tube on ice to dialyze for 1 h.
5. Centrifuge the samples at 13,000* g* for 10 min at 4 °C. Transfer the supernatant (S2, about 250 μl each tube) and combine with S1 tube, mix them on ice (500 μl in total each tube).

### Chromatin immunoprecipitation

1. Add 500 μl of incubation buffer into the mixed supernatants and gently invert to mix.
2. Prepare the Dynabeads® Protein A. Take 10 μl of dynabeads protein A and wash the beads by 500 μl incubation buffer. Then centrifuge the tube at 300* g* for 30 s at 4 °C and discard the supernatant. Repeat the wash at least three times.
3. Pre-clear the digested chromatin by adding 10 μl of dynabeads protein A (washed by incubation buffer in advance) into the tube. Incubate on a rotating wheel at 4 °C for 2 h.
4. Centrifuge the sample to pellet the beads at 300* g* for 30 s at 4 °C. Transfer the supernatant and discard the beads.
5. Take 90 μl of supernatant as input and divide the remaining fractions into two aliquots (about 450 μl each)
6. Add 2–5 μg of proper antibody (anti-H3K9me2, anti-H3K36me3 or anti-H3K9/K14ac in our case) into each tube for immunoprecipitation. Incubate the two tubes on a wheel with gentle rotation overnight at 4 °C.
7. Take the tubes from the rotating wheel, and centrifuge at 300* g* for 30 s at 4 °C to collect the solution both in the lid and wall of the tubes.
8. Add 30 μl of dynabeads protein A (washed by incubation buffer in advance) into each tube.
9. Incubate the tubes on the rotating wheel with gentle rotation at 4 °C for 6 h.
10. Centrifuge the tubes at 300*g* for 30 s at 4 °C. Put the tubes in the pureproteome magnetic stand on ice for beads separation from solution.
11. Remove the supernatant completely with pipette tip. Wash the beads with 1 ml washing buffer from low salt wash buffer to high salt wash buffer. Put the tubes on the rotating wheel at 4 °C for 5 min for each wash process. Centrifuge the tubes at 300*g* for 30 s at 4 °C each time when taking off from the wheel.
12. Pipette out as much the washing buffer as possible from the beads after the last wash.
13. Elute the immune complex from beads by adding 300 μl of preheated elution buffer (42 °C) in each tube. Incubate the tubes at 65 °C for 15 min. Invert the tubes every 5 min to mix the solution during incubation.
14. Centrifuge the tubes at 300*g* for 30 s at 4 °C and put the tubes in the pureproteome magnetic stand for a few seconds. Transfer the supernatants into new 2 ml Eppendorf tubes. Leave the beads on ice for next elution step.
15. Repeat step 13–14 by adding another 300 μl preheated elution buffer to the beads in each tube left from step 11.
16. Combine the two eluates (about 600 μl in total) from each sample, and add 510 μl of elution buffer to 90 μl of input solutions for DNA extraction.

### DNA extraction

1. Add equal volume of phenol/chloroform/isoamyl alcohol (25:24:1) to the eluates and input solutions. Vortex each tube briefly.
2. Centrifuge at 13,000*g* for 10 min at room temperature and transfer the supernatant (about 550 μl) into 2 ml Eppendorf tubes.
3. Add 2 μl of glycogen, 1/10 volume of 3 M sodium acetate (pH 5.2), 2.5 volume of 100% ethanol to each tube. Invert each tube gently for three times to mix and incubate at − 80 °C for 1 h.
4. Centrifuge at 13,000*g* for 10 min at 4 °C and discard the supernatant.
5. Wash the pellet with 500 μl of 70% ethanol. Invert the tube to wash off the solutions on the wall of each tube.
6. Centrifuge at 13,000*g* for 10 min at 4 °C and remove the supernatant.
7. Dry the pellet at room temperature for 10 min and add 50 μl of double distilled water into each tube to resolve the DNA.
8. Take 5 μl of dissolved DNA from each tube to perform quantitative real time PCR to check the pull-down efficiency, and store the left DNA in − 80 °C.

### Chromatin preparation by X-ChIP

A X-ChIP protocol which had been successfully applied in *Arabidopsis thaliana* [22] was performed for strawberry fruit tissues with minor modifications. Briefly, two grams of woodland strawberry fruit was cut into four equal parts and vacuumed for 10 min in 1% formaldehyde. A total of 500 μl of lysis buffer was used to dissolve the chromatin. Then the supernatant was sonicated for fragmentation by Bioruptor. The parameter of sonication was set as 15 s on and 45 s off for 4 cycles, which resulted in chromatin with DNA fragments ranging from 250 to 750 bp. Fragmented chromatin was diluted twice by incubation buffer and immunoprecipitated by the anti-H3K9me2 or the anti-H3K36me3 antibody.

### Quantitative real-time PCR to check the pull-down efficiency of immunoprecipitation

Quantitative real-time PCR was performed using SYBR Premix Ex Tag (Takara) in a volume of 20 μl, which contained 6.6 μl of ddH2O, 10 μl of SYBR Green Master Mix (Bio-Rad), 0.8 μl of each specific primer (10 μM), and 1.8 μl of diluted ChIP or input DNA template. ChIP DNA and the corresponding input DNA diluted by five-fold were used as template. The PCR reactions were carried out as follows: 95 °C for 3 min, 39 cylcles of 95 °C for 10 s and 55 °C for 30 s, 65 °C for 10 s. ChIP-qPCR data were analyzed to calculate the pull-down efficiency relative to input. The calculation was carried out consulting to Mendez FM [23].

### DNA quality check, library construction and sequencing

The concentration of immunoprecipitated DNA was checked by Qubit Fluorometer, Invitrogen. Sample integrity and purity were checked on Agilent Technologies 2100 bioanalyzer. After quality check, the ChIPed DNA was subjected to end-repair, 3′ adenylated and adding adaptors. Then PCR was performed for amplification and the PCR products were purified and selected (100–500 bp) with the Agencourt AMPure XP-Medium kit. Then the double stranded PCR products were heat denatured and circularized by the splint oligo sequence. The single strand circle DNA (ssCir DNA) were formatted as the final library. After quantified by Qubit ssDNA kit, the library was amplified to make DNA nanoball (DNB) for sequencing, and single end 50 bases reads were generated in the way of sequenced by combinatorial Probe-Anchor Synthesis (cPAS). The library amplification and sequencing were performed by The Beijing Genomics Institute (BGI).

### ChIP-seq data processing

The clean data in FASTQ format were mapped to the *Fragaria vesca v4.0* genome (https://www.rosaceae.org/species/fragaria_vesca/) by Bowtie2 [24, 25]. Then the SAM files were converted into BAM format and sorted by SAMTOOLS [26, 27]. Peak calling was performed based on the sorted BAM files by using MACS2 (-g 2.4e8 -q 0.05) [28]. The BAM files were also used for IGV visualization and Metagene analysis by Deeptools [29]. According to RNA-seq data in the red-stage fruits [30], expressed genes were evenly divided into five groups and genes without detectable transcripts were set as an additional group as well. The distribution of H3K9/K14ac in genic regions of the respective six gene groups was analyzed by Deeptools [29].

## Results

### Nuclei isolation from the strawberry fleshy fruit tissues

The procedure of nuclei isolation we performed was based on published protocols with some modifications [13, 19–21]. A comparison between our protocol and some other available N-ChIP protocols is summarized in Table 1. Strawberry fruits have high content of water, thus it took long time (usually 15–20 min at our hand) to produce fine powder by grinding in liquid nitrogen. Furthermore, the high level of polysaccharides and other secondary metabolites require repeated washes to collect clean and white nuclei from crude tissues. In our protocol, we used glass homogenizer for complete disruption of the fruit tissues, followed by further homogenization on ice with gentle shaking, and several washes of the cells by a large volume of nuclei isolation buffer. We recommend repeated washes at least four times using a large volume of nuclei isolation buffer before MNase digestion. In addition, we found that several washes by PBS after the liquid nitrogen step help to collect cleaner nuclei as well.

**Table 1:** Comparison between our protocol and some other available N-ChIP protocols

Procedures	Others [13, 19–21, 43–47]	Our optimized N-ChIP protocol
Starting material	Non-fruit tissues or suspension cells [13, 19–21, 43–47]	Fleshy fruit tissues
Nuclei isolation	No particular steps for homogenization [20, 21, 47]. Homogenize in an all-glass homogenizer using a tight pestle [13, 45, 46]. Homogenize in a roller stirrer [19] or through syringe [44]. Homogenize in an undescribed way [43]	The powder was washed in PBS-PSV twice firstly. Homogenize in a glass homogenizer followed by lysis on ice with gentle agitation for 10 min
Chromatin extraction	Extraction buffer contains 0.4–0.8% NP40 [45, 46], 0.1% Triton X-100 [43, 47], 0.5% Triton X-100 [21], 1% Triton X-100 [44], 0.25% Tween 40 [19], 1% Tween 40 [13] as the detergent, or no detergent [20]	Extraction buffer contains 0.5–1% Triton X-100 Wash at least four times
Fragmentation	Digestion at 37 °C for 10–12 min [19–21, 43, 46] or 4–7 min [19, 44, 45, 47]	Digestion for 10 min at 37 °C (30U/400 μl). Recommend optimizing the enzyme dosage and digestion time for each preparation
Immunoprecipitation Antibody Protease inhibitors Reducing reagents	Protein A–Sepharose [13, 19, 20, 45], Dynabeads Protein G [46], Dynabeads Protein A [21, 47] or Dynabeads Protein A/G beads [43, 44] ChIP-grade or only tested by western blot [13, 19–21, 43–47] Buffers contain protease inhibitor cocktail [20, 43–47], 0.1–0.2 mM PMSF [13, 19, 20, 43–45] Buffers contain 0.5 μM–0.5 mM DTT [45, 46], 10 mM β-mercaptoethanol [21, 43, 47]	Dynabeads Protein A beads Passed ChIP-grade antibodies validated by the ENCODE project Buffer contains 1 × protease inhibitor cocktail tablets, 1 mM PMSF Buffers contain 1 mM DTT and 11 mM β-mercaptoethanol

### Determination of a proper time for micrococcal nuclease digestion

**Fig. 2:**
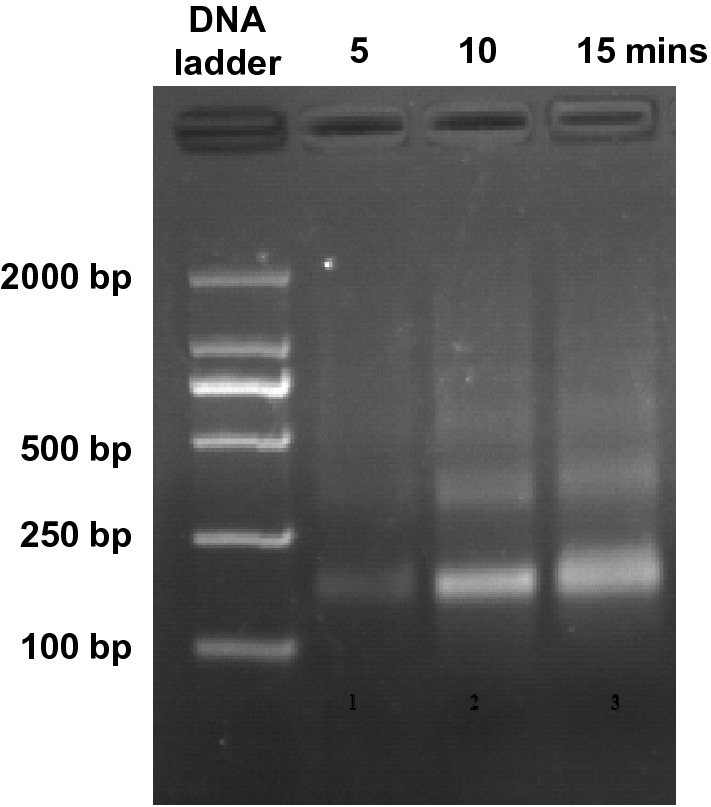
DNA fragmentation by micrococcal nuclease digestion. Chromatin samples were digested by 1 μl of micrococcal nuclease for 5, 10 and 15 min, respectively

A suitable size distribution of DNA fragments is crucial for ChIP. The efficiencies of micrococcal nuclease are affected by the amounts of DNA available in the reaction systems. Thus, the appropriate digestion time needs to be determined for each chromatin preparation. We checked the size distribution of genomic DNA on gel after 5, 10 and 15 min of micrococcal nuclease digestion, and found that large amounts of mononucleosomes were obtained after 10 min of digestion (Fig. 2). Thus, we performed 10 min of micrococcal nuclease digestion to produce a suitable size distribution with least digestion time for further immunoprecipitation.

### Test of the pull-down efficiency of N-ChIP by qRT-PCR assays

**Fig. 3:**
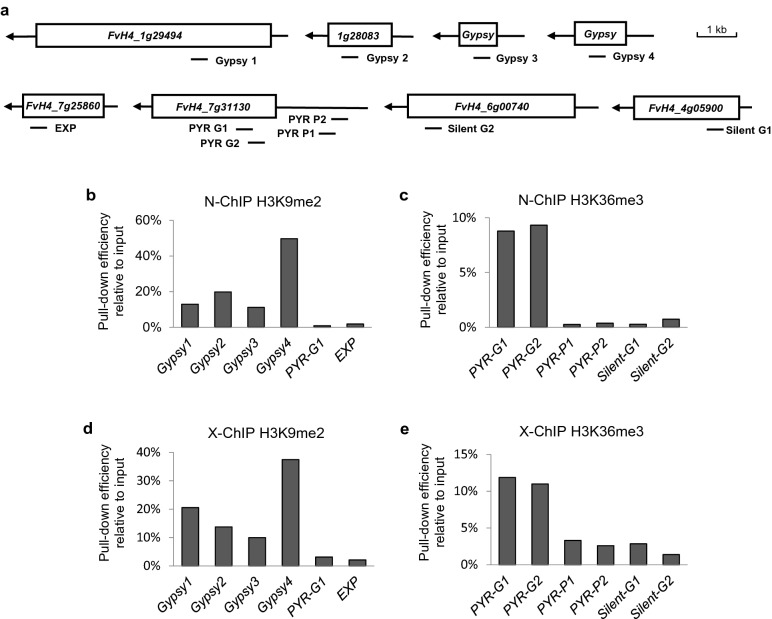
The pull-down efficiency of N-ChIP and X-ChIP methods for strawberry fleshy fruit tissues. **a** The DNA fragments profiled by qRT-PCR to check the pull-down efficiency and relative enrichment of ChIP experiments. (b-c) The pull-down efficiencies and the relative enrichments of H3K9me2 (**b**) and H3K27me3 (**c**) by our N-ChIP method. **d**, **e** The pull-down efficiencies and relative enrichments of H3K9me2 (**d**) and H3K27me3 (**e**) by the X-ChIP method

To check the quality of the fragmented chromatin and the pull-down efficiency of immunoprecipitation, qRT-PCR assays were performed to measure the enrichment levels of those histones marks of interest. Dimethyl-H3-lysine 9 (H3K9me2) is a silent mark typically enriched in transposable elements (TEs) and heterochromatic regions [31, 32]. Thus, four LTR fragments (named Gypsy 1, Gypsy 2, Gypsy3 and Gypsy 4) were profiled as the positive loci, while the gene bodies of two highly expressed euchromatic genes (fragment EXP and PYR-G1) were used as the negative control loci (Fig. 2a). Trimethyl-H3-lysine 36 (H3K36me3) is an active mark enriched in the gene bodies of transcribed genes [33]. Thus, two fragments within the gene body of the highly expressed *PYR* gene (fragment PYR-G1 and PYR-G2) were designed as the positive loci; two fragments in the promoter of *PYR* (fragment PYR-P1 and PYR-P2) and two fragments in the gene bodies with low levels of transcription (gene *HAC1* and *NCED2,* fragment silent-G1 and silent-G2), were designed as the negative controls (Fig. 2a). The primer sequences are listed in Table 2. The relative enrichment was calculated as previously described [23]. Two biological replicates were performed for each histone mark, which gave similar results. Thus, statistics from one replicate were shown in figures (Fig. 3b–e).

**Table 2:** Primer sequences used in ChIP-qPCR assays

Primer names	Gene ID	Primer sequence (5′ → 3′)
Gypsy 1_F	FvH4_1g29494	CCTGTGGGACACTCACATTC
Gypsy 1_R	FvH4_1g29494	GGAAGGGACAAGTTAGGAGC
Gypsy 2_F	FvH4_1g28083	AGATCATGTCCTTTGAGGAGC
Gypsy 2_R	FvH4_1g28083	CTTCAAGTGAGGACTTCGGT
Gypsy 3_F	–	TTAACCCATTTCGACCCTATTT
Gypsy 3_R	–	GGCTTCATTATGTTGCCTTTAC
Gypsy 4_F	–	AGAGACCAGCATGCAAACCTTA
Gypsy 4_R	–	TATGGCGGCACGTGGTAGTG
EXP_F	FvH4_7g25860	CACTCGGTTTCGATCAAAGG
EXP_R	FvH4_7g25860	TGACCTGAAAAGACAGGGCT
PYR-G1_F	FvH4_7g31130	GGAGGTGCGAGTGAGTACATT
PYR-G1_R	FvH4_7g31130	CGCTTTGATGTGCCTGACG
PYR-G2_F	FvH4_7g31130	GGGAATAATAAGAATGGAGGAGGA
PYR-G2_R	FvH4_7g31130	GAGCGTAGAGCTACACTGATG
PYR-P1_F	–	GTGCGACATAGATAACCGGA
PYR-P1_R	–	ACCTCATGTGTGATTTTACTGAA
PYR-P2_F	–	TGGCCTTTGCACATATAACACT
PYR-P2_R	–	CAAACTGAAACGAGTTGGAAGT
Silent-G1_F	FvH4_4g05900	TCCTATGTTTACGCCGTTGG
Silent-G1_R	FvH4_4g05900	GCGGGTGTACCTACAAGAATAG
Silent-G2_F	FvH4_6g00740	CTGAAGGATTGAGTGCAGAATTG
Silent-G2_R	FvH4_6g00740	CAGTTTCGGTTCGGGTAAGA

As shown by our qRT-PCR results, for both ChIPs against H3K9me2 and H3K36me3, the positive loci have higher enrichment levels relative to the negative loci (Fig. 3b, c). For the immunoprecipitation against the silent mark H3K9me2, the pull-down efficiency of the four Gypsy loci ranged from 11.2 to 49.7% (relative to input), much higher than the negative loci which ranged from 0.8 to 1.8% (Fig. 3b). For the active mark H3K36me3, the pull-down efficiency in the active gene body ranged from 8.8 to 9.3%, higher than the promoter region (0.26–0.38%) or the silent gene body (0.28–0.74%) (Fig. 3c). Thus, the chromatin produced by our N-ChIP protocol has a high pull-down efficiency for both active and silent histone marks when strawberry fleshy fruit tissues are used.

### Comparison of the pull-down efficiency between N-ChIP and X-ChIP for strawberry fruit tissues

X-ChIP is widely used for profiling histone modifications in both plants and animals [22, 34]. To investigate whether N-ChIP is a more suitable protocol for strawberry fruits relative to X-ChIP, we further performed X-ChIP in strawberry to study how it worked. Immunoprecipitation was done with anti-H3K9me2 and anti-H3K27me3 antibodies and the quality of the chromatin was checked by qRT-PCR as well.

For X-ChIPs against both H3K9me2 and H3K36me3, the enrichment levels for the positive loci were higher than the negative loci. Specifically, the pull-down efficiency for the enriched loci ranged from 11.0 to 37.5% (the four Gypsy loci) and 11.0 to 11.8% (the active gene body) for H3K9me2 and H3K36me3, respectively, which is comparable to N-ChIP (Fig. 3d, e). On the other hand, the pull-down efficiency of the depleted loci ranged from 2.1–3.1% and 1.4–2.9% for H3K9me2 and H3K36me3, respectively, which was higher than N-ChIP (Fig. 3d, e). Thus, compared to N-ChIP, the X-ChIP protocol results in a higher background noise and a lower signal-to-noise ratio when strawberry fleshy fruit tissues are used.

### Determination of the consistency and reproducibility by sequencing

To test the consistency and reproducibility of our N-ChIP method, the genome-wide distribution of histone acetylation (by anti-H3K9/K14ac) in the red-stage strawberry receptacles was profiled by sequencing, and two biological replicates were performed.

**Fig. 4:**
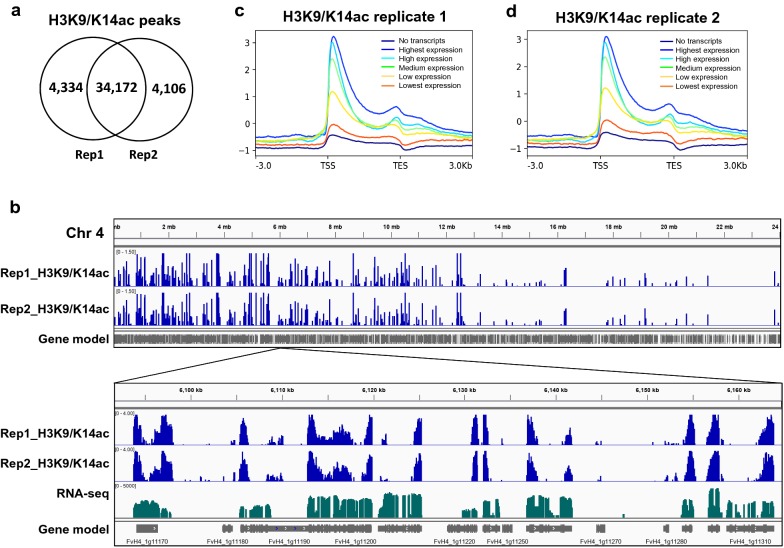
The consistency and reproducibility of our N-ChIP protocol validated by ChIP-seq profiles. **a** A venn diagram showing the overlap between the H3K9/K14ac-enriched peaks called by ChIP-seq replicate 1 and 2. **b** IGV browser shots illustrating chromosome-wide and local H3K9/K14ac enrichments for the two biological replicates. **c**, **d** Metagene analysis illustrating a positive correlation between local H3K9/K14ac enrichment and gene expression levels. The expressed genes were evenly classified into five groups according to their transcript levels (blue-highest expression, light blue-high, green-medium, yellow-low and red-lowest). The group of genes with no detectable transcripts was included as well (black line). The transcriptome data were published previously [30]

Overall, 38,506 and 38,278 H3K9/K14ac enriched peaks were detected in the two biological replicates, respectively (Fig. 4a). Among those enriched peaks, 34,172 are overlapped (88.7% and 89.2% for replicate 1 and replicate 2, respectively), which indicates a highly consistent genome-wide distribution between the two biological replicates.

In addition, the two N-ChIP-seq replicates produce a very similar enrichment profiles both chromosome-wide and in a local view (Fig. 4b). Overall, H3K9/K14ac is enriched in the gene-dense euchromatic region while depleted in the gene-parse heterochromatic region (Fig. 4b). Locally, H3K9/K14ac is highly enriched around the TSS (transcription start site) region of the transcribed genes (Fig. 4b–d).

Histone acetylation is a typical active euchromatic mark indicative of transcription in animals and plants, e.g.* Arabidopsis* [35, 36]. To investigate whether this active role of histone acetylation on transcription is conserved in woodland strawberry, we partitioned the transcribed genes evenly into five groups according to their magnitude of transcription [30], and profiled the enrichment level of H3K9/K14ac. We found that in the red-stage strawberry receptacles, gene expression levels were positively correlated with the H3K9/K14ac enrichment levels (Fig. 4c, d), which indicated an active role of histone acetylation in strawberry as well. In sum, our N-ChIP procedure produces highly consistent ChIP-seq data for histone acetylation which is in accordance with its regulatory role in gene expression and chromatin structure.

## Discussion

Here we describe a modified N-ChIP method suitable for profiling histone modifications in strawberry fleshy fruit tissues. Test of the quality of chromatin and the efficiency of immunoprecipitation by qPCR demonstrates a reasonably high pull-down efficiency and low levels of background signals for both active and silent histone marks by our protocol. In addition, the consistency and reproducibility of our protocol were confirmed by the high-throughput sequencing data, in which nearly 90% of the enrichment peaks were overlapped between biological replicates.

The key steps for a reliable ChIP protocol for strawberry fruits include the isolation of clean nuclei, an efficient way to exclude protease and secondary metabolites during chromatin preparation, a proper DNA size distribution after fragmentation, a ChIP-grade antibody and a proper way to test the efficiency of immunoprecipitation [13, 14, 37, 38]. Unlike the cells collected from suspension cultures, fruit tissues have rigid cell wall, large amounts of polysaccharides, large vacuoles, high content of water and large amounts of secondary metabolites such as phenolics. We recommend applying glass homogenizers to completely separate the individual cells as much as possible by physical ways followed by further homogenization on ice. Multiple washes with a large volume of PBS and nuclei isolation buffer help to wash away the metabolites and collet clean nuclei. To exclude protease and secondary metabolites during chromatin preparation, we recommend washing the cell lysate by PBS after homogenization, and applying multiple protease inhibitors during preparation of chromatin. For the choice of antibody, we recommend taking advantage of those published works on the quality assessment of the histone modification antibodies, which screen the commercially available ChIP-grade antibodies by western blotting and ChIP-array or ChIP-seq with strict criteria [39]. In addition, we recommend the primers suitable for testing the efficiency of immunoprecipitation against the active mark H3K36me3 and the silent mark H3K9me2 for further research. We suggest that this method could be used in tissues with high water content, high levels of polysaccharides and phenolics in other plant species.

X-ChIP is widely used for profiling histone modifications in both plants and animals [22, 34], which has been applied in tomato, strawberry and other fleshy fruits as well [40, 41]. We have two lines of evidence indicating that our N-ChIP protocol performs better than X-ChIP when strawberry fruit tissues are used. Firstly, although the pull-down efficiency of N-ChIP and X-ChIP are comparable, N-ChIP has a higher signal-to-noise ratio than X-ChIP. In our N-ChIP, the fold enrichment of the positive loci relative to the negative loci ranges from 6.1- to 58.9-fold for H3K9me2 and 11.9- to 35.9-fold for H3K36me3 (Fig. 3b, c). While in X-ChIP, the fold enrichment ranges from 3.2- to 17.6-fold for H3K9me2 and 3.3- to 8.5-fold for H3K36me3 (Fig. 3d, e), which is lower than N-ChIP. Secondly, N-ChIP has a lower non-specific pull-down for the depleted loci, which is of great importance to the accuracy and reliability of peak calling in the data analysis for the following sequencing (Fig. 3). The higher background level might be caused by the cross-linking process. It is not unexpected since cross-links by for-maldehyde may lead to the identification of indirect or transient protein-DNA interactions, particularly in highly transcribed regions [13, 42].

Obtaining the genome-wide distribution of histone modifications is of great importance for understanding its possible regulatory role in chromatin regulation. However, producing highly consistent enrichment profiles among biological replicates is challenging in ChIP-seq experiments. Our sequencing results validate the consistency and reproducibility of the N-ChIP method we applied. For the histone mark H3K9/K14ac, as high as 88.7% and 89.2% of the called peaks are overlapped by the two biological replicates, respectively. In addition, the two ChIP-seq replicates produce consistent enrichment profiles both chromosome-wide and in a local view demonstrated in the metagene analysis. In a chromosome-wide view, we show that H3K9/K14ac is enriched in the gene-dense euchromatic region while depleted in the TE-rich heterochromatic region (Fig. 4b). Locally, H3K9/K14ac is highly enriched around the TSS (transcription start site) region of the transcribed genes (Fig. 4b-d), and positively correlates with the levels of gene transcripts, which is consistent with the active role of histone acetylation. In sum, both qPCR and high-throughput sequencing data suggest the reliability and consistency of our N-ChIP procedure, which have not been validated for X-ChIPs in fleshy fruits.

## Conclusions

In this study, we report an optimized N-ChIP method for strawberry fruit tissues to achieve an efficient pull-down, a high signal-to-noise ratio and the highly reproducible genome-wide profiles by high-throughput sequencing for the histone modifications of interest. Our work lays a foundation for the study of histone-related chromatin structure in fleshy fruits and potentially in other fruits with high levels of polysaccharides for the community.

## Data Availability

The datasets used and/or analyzed during the current study are available from the corresponding author upon request.
